# Accurate flow in augmented networks (AFAN): an approach to generating three-dimensional biomimetic microfluidic networks with controlled flow

**DOI:** 10.1039/c8ay01798k

**Published:** 2018-12-03

**Authors:** Jiaming Guo, Keely A. Keller, Pavel Govyadinov, Paul Ruchhoeft, John H. Slater, David Mayerich

**Affiliations:** a Department of Electrical and Computer Engineering , University of Houston , USA . Email: mayerich@uh.edu; b Department of Biomedical Engineering , University of Delaware , USA

## Abstract

*In vivo*, microvasculature provides oxygen, nutrients, and soluble factors necessary for cell survival and function. The highly tortuous, densely-packed, and interconnected three-dimensional (3D) architecture of microvasculature ensures that cells receive these crucial components. The ability to duplicate microvascular architecture in tissue-engineered models could provide a means to generate large-volume constructs as well as advanced microphysiological systems. Similarly, the ability to induce realistic flow in engineered microvasculature is crucial to recapitulating *in vivo*-like flow and transport. Advanced biofabrication techniques are capable of generating 3D, biomimetic microfluidic networks in hydrogels, however, these models can exhibit systemic aberrations in flow due to incorrect boundary conditions. To overcome this problem, we developed an automated method for generating synthetic augmented channels that induce the desired flow properties within three-dimensional microfluidic networks. These augmented inlets and outlets enforce the appropriate boundary conditions for achieving specified flow properties and create a three-dimensional output useful for image-guided fabrication techniques to create biomimetic microvascular networks.

## Abstract 2

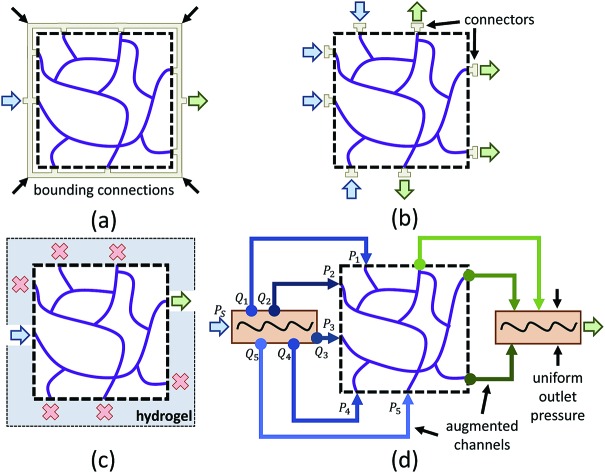
A network augmentation approach that adds synthetic connections to microvascular networks to induce biomimetic microfluidic flow.

## Introduction

The ability to generate vascularized tissue constructs has been a major challenge in the field of tissue engineering for decades.^1^–^3^ To overcome this limitation, small volume constructs have been implemented that rely on diffusion-mediated transport to deliver oxygen, nutrients, and soluble factors, and to remove waste products. The ability to generate hydrogels laden with microvasculature that recapitulates the dense and tortuous architecture of *in vivo* vascular networks could aid in fabricating larger volume tissue constructs^4^ and advanced cell culture platforms to model physiological and pathological processes.

Two general approaches to generate vascularized constructs have been developed: self-assembly of networks by vascular cells and formation of pre-defined, engineered vasculature followed by vascular cell seeding. Self-assembly provides a straightforward approach to create microvasculature in both natural^5^–^9^ and synthetic^10^,^11^ hydrogels. When performed in hydrogels housed in a microfluidic device, self-assembled networks can anastomose with larger microfluidic channels allowing for fluid flow through the networks.^5^,^8^,^12^ This approach has been implemented to investigate leukocyte adhesion to vessel walls,^8^ cancer cell extravasation,^13^ convective transport,^10^ soluble factor signaling during vasculogenesis,^14^ and endothelial cell response to fluid flow.^8^ While self-assembly is a powerful approach to generate microvascular networks, there is no control over the final architecture, therefore making it impossible to use the same network across multiple experiments or to easily couple experimental data with computational fluid dynamics (CFD) models. Furthermore, self-assembly has not been implemented to generate larger diameter arterioles and arteries needed to reproduce the hierarchical structure of *in vivo* vasculature.

To overcome these limitations, various biofabrication techniques have been developed that allow pre-definition of network architecture prior to seeding with vascular cells. Many of these techniques are amenable to generating two-dimensional (2D), planar networks embedded in hydrogels which have been implemented for drug screening,^15^,^16^ blood flow modeling,^17^–^19^ disease modeling,^20^,^21^ to investigate flow-mediated signaling between tumor and endothelial cells,^22^ cancer cell extravasation,^23^ self-healing of vasculature post inflammation,^24^ and thrombotic response of vessels due to inflammation.^20^ While many existing microfabrication approaches allow for direct control over 2D, planar networks, they do not duplicate the complex 3D structure of *in vivo* networks.^25^ A few techniques to generate 3D, non-planar networks have been developed, including 3D printing of sacrificial carbohydrate glass,^26^ modular assembly,^27^ and 3D bio-printing.^28^ These approaches have been implemented to increase cell viability in larger volume constructs,^26^ to create a perfusable microfluidic hydrogel,^27^ and to spatially organize both cells and vasculature in a tissue construct.^28^ Although these approaches allow repeated fabrication of 3D vascular networks with well-defined geometry, they are incapable of fabricating dense and tortuous small-diameter structures needed to recapitulate *in vivo* microvascular architecture. Direct-write assembly^29^ and omnidirectional printing,^30^ are capable of generating a hierarchical vascular system with a wide range of diameters, 10–530 μm for direct-write assembly and 18–600 μm for omnidirectional printing, but are not yet amenable to generating dense, tortuous, and highly interconnected microvascular networks. To overcome this problem, we recently developed an image-guided, laser-induced hydrogel degradation (LIHD) technique that utilizes either computer aided design (CAD) synthetic networks or 3D image stacks of *in vivo* vasculature as digital templates to fabricate 3D, biomimetic, hydrogel-embedded microfluidic networks whose architecture closely matches that of *in vivo* microvasculature.^31^,^32^ Laser-induced degradation is amenable to both synthetic and natural hydrogels^31^–^39^ and has been utilized to generate microfluidic networks in cell-laden constructs.^40^

**Fig. 1:**
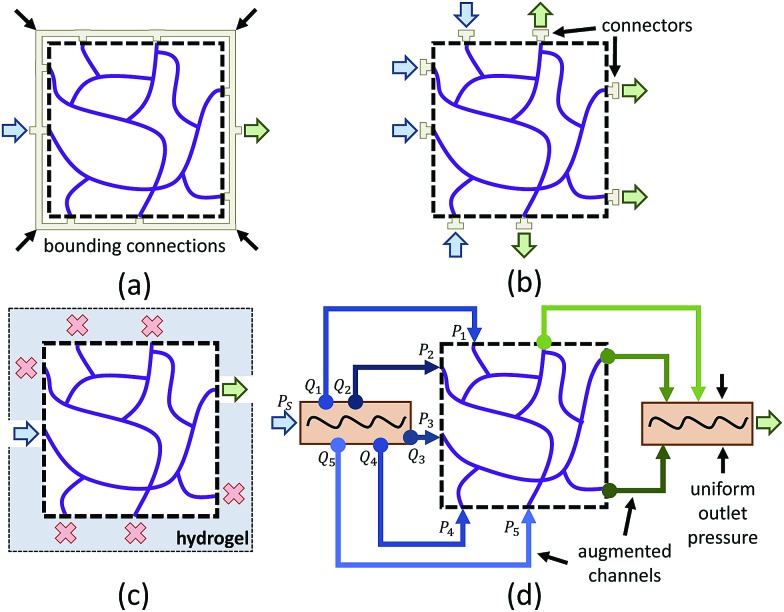
An illustration demonstrating the challenges for addressing boundary conditions in microfluidics-based microvascular models (a–c), along with our solution (d). (a) A single-inlet, single-outlet network created by adding a bounding channel that connects all dead-ends. As there is no direct control over the bounding connections, the resulting model exhibits irregular flow. (b) A multi-inlet, multi-outlet network created by attaching a series of peripheral pumps to provide necessary boundary conditions, which is impractical for 3D, non-planar networks. (c) A single-inlet, single-outlet network effectively eliminates flow through a majority of branches by blocking most terminations (red X's). (d) Our solution constructs connections between network terminations and pre-defined feeders to enforce the appropriate boundary conditions and thus desired flow properties in both 2D and 3D networks.

One limitation of using 3D image stacks of *in vivo* vasculature as a digital template is the presence of dead-end vascular structures that impede flow (Fig. 1c). Similar to modeling synthetic microvascular networks (SMNs), real microvascular networks (RMNs) must be cropped to fit within designed volumes. Since RMNs exhibit a high degree of connectivity, the selection of any region results in an incomplete network with multiple terminations, making it difficult to prescribe desired boundary conditions. Two generalized methods have been proposed to overcome this problem: incorporating multiple inlets and outlets to the network (Fig. 1b)^41^ or completing the network with additional connections to force the terminating vessels to converge to a single bounding channel (Fig. 1a).^40^ While the first approach is capable of generating physiologically realistic flow through the network,^42^ it is limited to 2D, planar networks. This approach also requires the implementation of multiple syringe pumps and connectors which is experimentally cumbersome. The second method (Fig. 1a) offers a simple solution to this problem by adding an additional bounding channel that connects all of the network dead-ends to a single inlet and outlet, but has only been implemented for 2D, planar networks and does not provide control over which dead-ends are inlets or outlets, thereby limiting control over flow properties. To overcome these problems, we developed a network design approach that constructs synthetic connections between boundary nodes and two feeders (Fig. 1d), an inlet and outlet, allowing for well-controlled boundary pressure, at previous dead-ends based on established models or *in vivo* measurements. Accurate flow simulations are required to calculate these boundary connections. Rigorous numerical methods, such as the finite element method^43^ and finite difference method^44^ are frequently used in CFD. However, determining the parameters for the necessary augmented elements would require a time-consuming iterative optimization step that is impractical on most workstations. Accordingly, we performed this optimization using the linear Hagen–Poiseuille (H–P) method, which allows for a rapid extrapolation of pressure-driven flow through fabricated networks. We implemented these features in an interactive software package called Accurate Flow in Augmented Networks (AFAN) using C++ and CUDA for fast evaluation and real-time visualization.

We demonstrate that the resulting augmented network design can be fabricated using LIHD. We also demonstrated that our augmented microvascular model has a high level of flow predictive capability based on comprehensive CFD models and microfluidic experiments. This approach lays the foundation for implementing 3D image stacks as digital templates for fabrication of biomimetic vascular networks embedded in hydrogels whose architecture and flow properties accurately mimic *in vivo* vasculature.

## Materials and methods

### Two-dimensional Wheatstone bridge network

We designed and fabricated a microfluidic network based on the Wheatstone bridge using the AFAN user interface. Based on user input describing network architecture and flow properties, AFAN generates a binary mask used as the basis for fabrication.

The fabricated network was constructed based on our previously published protocols.^32^ Plasma-bonded polydimethylsiloxane (PDMS) and glass microfluidic devices were fabricated to provide connections for a syringe pump. For hydrogel incorporation within a device, devices were functionalized by washing with 2,2-dimethoxy-2-phenylacetophenone, and 3-(trimethoxysilyl)propyl methacrylate, to allow hydrogel bonding to the PDMS and glass.^10^ A pre-polymer solution of 5% weight per volume 3.5 kDa poly(ethylene glycol) diacrylate (PEGDA), 3.7 mM Alexa Fluor 633-labeled acryl-poly(ethylene glycol), and 10.2 mM lithium phenyl-2,4,6-trimethylbenzoylphosphinate (LAP) in HEPES-buffered saline (HBS) (pH 8.3, 10 mM HEPES, 100 mM NaCl) was photopolymerized inside the microfluidic device *via* exposure to UV light at 6 mW cm^–2^ for 30 seconds. A photo-mask placed in the light path was used to define the geometry of the hydrogel within the device. LIHD was used to create a microfluidic network in the PEGDA hydrogels as previously described.^31^–^33^ A series of virtual masks^45^ defining ROIs in *x*, *y*, and *z*, were generated to guide the position of a 140 fs pulsed Ti:S laser operating at 790 nm at 37.7 nJ μm^–2^ focused through a 20× (NA = 1.0) water immersion objective for selective hydrogel degradation.

The fabricated network was filled with 2000 kDa FITC-labeled dextran at 1 mg mL^–1^ in HBS and imaged *via* structured illumination. Particle image velocimetry was used to quantify the average velocity in each segment of the microfluidic network. Using a syringe pump, 3 μm diameter polystyrene spheres, at 8.4 × 10^6^ particles per mL in HBS, were flowed through the network at 25 μL h^–1^. Images of the particles were acquired using a 2 ms image acquisition time over 3 min intervals. An average of 200 particles per 3 min interval were analyzed per segment in triplicate. The center-to-center distance traveled by each particle was measured using ImageJ and the particle velocity was calculated by dividing the distance traveled by the 2 ms acquisition time. Particles that spanned segments, or that overlapped, were excluded and particle velocities were averaged for each segment. Constant fluid flow was maintained and verified throughout the image collection period. The particle streaks collected are colored to help distinguish particles from each other (Fig. 6b).

### Three-dimensional microvascular network

**Fig. 2:**
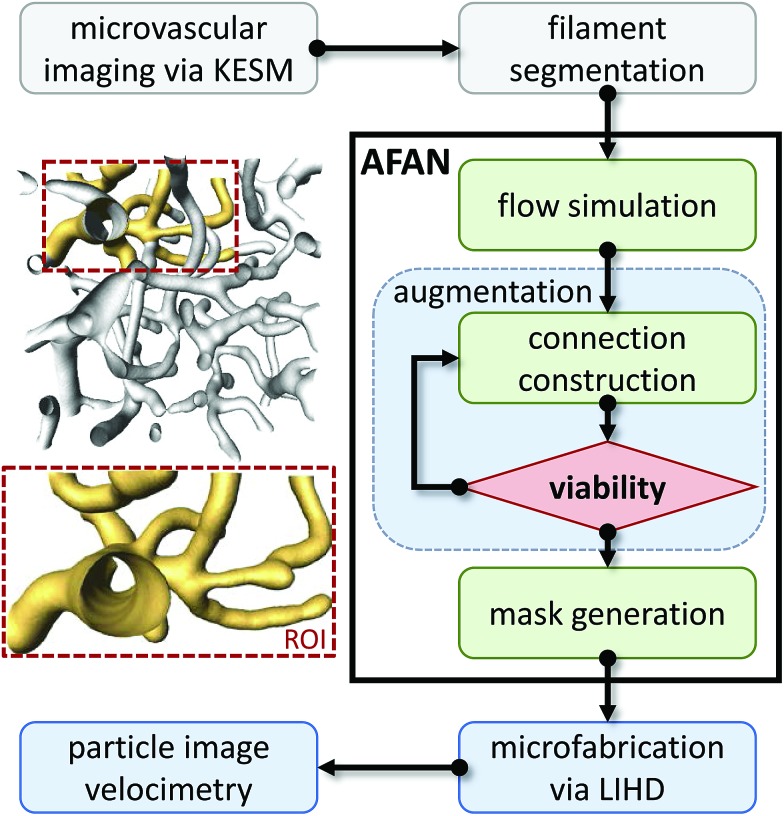
Overview of our method. An inset shows a viable ROI network culled from a larger microvascular network for augmentation and fabrication.

The overview for our approach is shown in Fig. 2. We used a whole mouse brain microvascular data set (Fig. 7a) collected using knife-edge scanning microscopy (KESM)^46^,^47^ available through the KESM Brain Atlas (; https://www.kesm.cs.tamu.edu).^48^ A 652 × 652 × 100 pixel (120 × 120 × 100 μm) region of interest (ROI) was identified and extracted from the whole-brain data set. Microvessel centerlines and connectivity were segmented using a predictor–corrector algorithm,^49^ while the surface model and radii were extracted manually by setting a threshold to separate the microvascular structure from the background. This data was combined to generate a graph-based model used as input to the AFAN software.

The AFAN user interface was used to specify boundary pressures producing flow velocities based on *in vivo* measurements.^50^,^51^ The AFAN H–P method was used to extrapolate flow properties throughout the ROI. The segmented network was then augmented with connections to enforce the desired boundary conditions. The augmented network was constructed such that an input volumetric flow rate *Q*_i_ produced the desired flow characteristics in the ROI (Fig. 1d).

A comparative CFD model was generated from an AFAN-produced binary mask and imported into OnShape (https://www.onshape.com), an online CAD package. The geometric mesh was constructed using a built-in meshing function in SimScale (; https://www.simscale.com), which creates polyhedral meshes for fluid-based models. SimScale was used to integrate an incompressible steady laminar fluid flow into the simulation with the simpleFoam solver in the OpenFOAM toolbox.^52^ We specified the appropriate materials and boundary conditions (inlet velocity, outlet pressure, and *no slip* walls), and conducted simulations on the cloud. The results were visualized and analyzed using ParaView,^53^ which offers a host of post-processing operations for data analysis. The augmented, 3D biomimetic network was fabricated in PEGDA hydrogels and visualized with fluorescent dextran in the same manner as the 2D network.

## Results and discussion

### Network characterization

Characterizing the hydraulic resistance of each synthetic connection relies on accurate boundary measurements of either boundary pressures or flow rates. The proposed interpretation of pressure-driven flow through circular microchannels uses the H–P equation based on boundary pressures:1
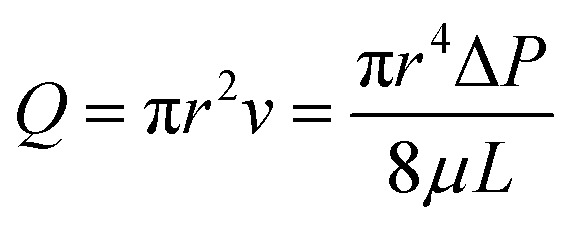
which estimates the volumetric flow rate *Q* as a function of the pressure drop Δ*P*, dynamic viscosity *μ*, channel length *L*, and channel radius *r*. If necessary, the corresponding wall shear stress *τ* can be calculated from the computed average flow velocity *v* based on eqn (2).2
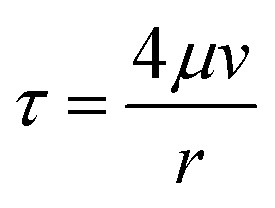



The H–P equation was initially derived for channels that are infinitely long with no variation in geometry, but microvessels often have finite lengths and changing cross-sections. Since the H–P method builds on several assumptions, including Newtonian, incompressible, and laminar flow properties, and boundary conditions such as the uniform pressure gradient condition, we provide the following justification to demonstrate its viability in our proposed models: for flow regimes, *in vivo* blood flow exhibits a Newtonian pattern when the shear rate is greater than or equal to 100 s^–1^,^54^ and small microvessels such as capillaries and arterioles satisfy this condition.^55^ Furthermore, fluids in capillaries are well approximated as incompressible mediums as the Mach number Ma drops below 0.2.^56^ Finally, the Reynolds number Re in capillaries is usually much smaller than 2000, and often less than 1, therefore such flow is completely laminar. As for boundary conditions, the H–P equation can be reasonably applied to channels with finite lengths if the fluid has a local and fully developed laminar flow profile.^57^ While a modified H–P equation was developed to correct for the tortuous and fractal properties of capillaries,^58^ a capillary fiber can also be approximated as a straight cylinder of the same length with no variation in diameter since inertial effects are negligible and the cross-sectional velocity is stable for curved paths in low Reynolds situations.^59^ In conclusion, the H–P method can be applied to extrapolate accurate pressure-driven flow in our proposed models.

Nevertheless, eqn (1) only quantifies the average velocity through one channel, requiring an integral method that links all channels together to solve for the entire network. A common technique is to apply an electric–hydraulic analogy using the following strategy:^57^

(1) Transform the hydraulic network into an equivalent electric circuit and complete the circuit with voltage sources (resembling pressure sources) based on an educated guess or previous measurements.

(2) Calculate and label each resistor based on eqn (1).

(3) Write an Ohm's function for each fiber and a Kirchhoff's current function^57^ for each branching point.

(4) Organize these equations into a linear system and solve with standard matrix factorization.

The high-dimensional matrix factorization step can take hours using CPU-based implementation for large networks. In this work, this step was performed using the CUBLAS library in combination with an nVidia Geforce 970 GTX graphics card to provide real-time performance, and interactive visualization and refinement of the network and its augmented components.

### Network augmentation

Since modeling entire microvascular networks is experimentally impractical, current studies rely on selected ROIs that are culled from a larger network. The peripheral network of an ROI plays an important role in regulating its boundary pressure. Simply building a microfluidic model consisting of an extracted ROI will not produce predictable flow as most boundary nodes are either open to atmosphere or abruptly terminated, and multiple source supplies can be attached to provide the necessary boundary conditions. However, this method is impractical for 3D networks that have vessels terminating from all directions of the ROI volumes. We addressed this by developing a network augmentation technique that connects all the inlet nodes to an inlet feeder and all the outlet nodes to an outlet feeder using synthetic connections. Not only do these connections compensate for the absence of the peripheral network in controlling boundary conditions, but they also reduce the number of sources connected to a microfluidic device and therefore can be easily integrated into most microfluidic devices.

The augmented network is composed of three parts: an ROI network, augmented connections, and two main feeders. The synthetic connections play the role of a flow splitter to simultaneously achieve the appropriate boundary pressure *P*_i_ at each end while keeping the ROI network unchanged (Fig. 1d). Since augmenting outlets is identical to inlets, we will focus our discussion on inlets in the following sections.

**Fig. 3:**
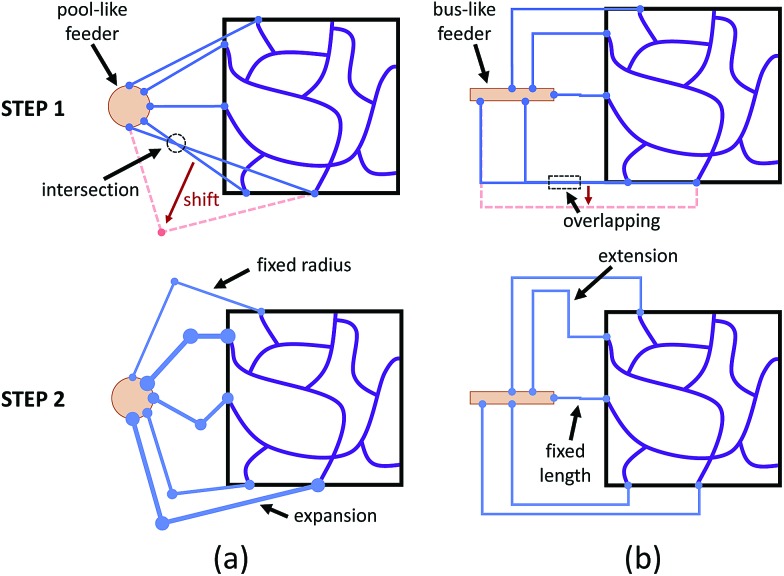
Augmenting networks with synthetic connections. (a) For planar networks, the *pool*-like feeders (orange circle) and direct connections are placed and arranged to avoid intersections. After fixing one channel radius, the rest of the connections are expanded to satisfy pressure constraints. (b) For non-planar networks, the *bus*-like feeders (orange rectangle) are placed and connected using homogeneously axis-aligned paths without overlap. After fixing one channel length, the rest of the connections are extended to satisfy pressure constraints.

According to eqn (1), building a synthetic connection with the appropriate hydraulic resistance requires a trade-off between channel length and radius. For planar networks, we fixed channel length and solved for radius as an easy way to avoid intersection (Fig. 3a). There are several graphical user interface libraries, such as the OpenGL library, that simplify 2D spatial arrangements. To build fixed-length connections, we introduced a pool-like feeder (a cylinder) with uniform pressure outputs around the surface. To obtain the source pressure, the inlet corresponding to the lowest source pressure was constrained to 5 μm. We expanded other connections, decreasing their resistance to match the calculated source pressure.

**Fig. 4:**
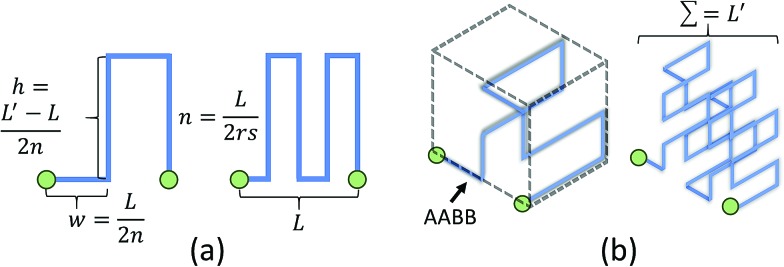
Two iterations of 2D and 3D square curves. (a) The total channel length *L*′ of a 2D square curve is computed by equation: *L*′ = *L* + 2*hn*. (b) The total channel length *L*′ of a 3D square curve is computed by equation: *L*′ = (1 + 2*n*)*L* + 4*hn*^2^.

For non-planar networks, we introduced a bus-like feeder (a cuboid) with uniform pressure outputs at the top and bottom faces (Fig. 3b), which confines connections to a single plane (*e.g. x*–*y*). This helps determine optimal arrangements while avoiding overlaps. To obtain the source pressure, we set all radii to 5 μm and fixed the length of the inlet requiring the highest source pressure. Other connections were extended to increase their resistance to satisfy the established boundary conditions, or calculated source pressures. Unlike channel expansion, channel extension must account for channel distribution and space utilization. While multiple methods can be used to increase the channel length, one can simply replace the original connections with longer straight connections. Unfortunately, this approach sacrifices space efficiency for simplicity and may make the network difficult to fabricate. More complex paths, such as Hilbert curves,^60^ provide an efficient use of space, but are incompatible with our fabrication method as our hydrogels may not be able to mechanically support such a densely-packed structure. We address this by adopting a space-filling method using square curves (Fig. 4), which provides a compromise between simplicity, space efficiency, and fabrication integrity. Each of the square wave-like connections was constructed inside of a user-defined axis-aligned bounding box (AABB), which allowed for rapid testing of intersections and overlaps. The workflow to construct these connections is summarized as follows:

(1) Construct initial connections and arrange to avoid overlap.

(2) Optimize the curve order, *n*, based on an input parameter, *s*, used to control the channel sparsity.

(3) Optimize the channel width, *w*, and channel length, *h*, based on the desired total channel length, *L*′.

(4) Build an AABB for each connection and check for intersections using AABB collision detection algorithms.

Refinement of square curves and other interactive functions have been incorporated into AFAN. Microvessels were rendered as truncated generalized cones (TGCs)^61^ defined by two adjacent points along a fiber that outline the local vessel shape and diameter (Fig. 7d). Volumetric flow and pressure were interactively calculated and visualized using arrow glyphs color-mapped by flow speed. A group of keyboard and mouse commands are registered for interactive purposes, allowing users to create customized augmented channels attached to an RMN. Conveniently, boundary pressure values are specified with only a few clicks.

### Two-dimensional Wheatstone bridge network

**Fig. 5:**
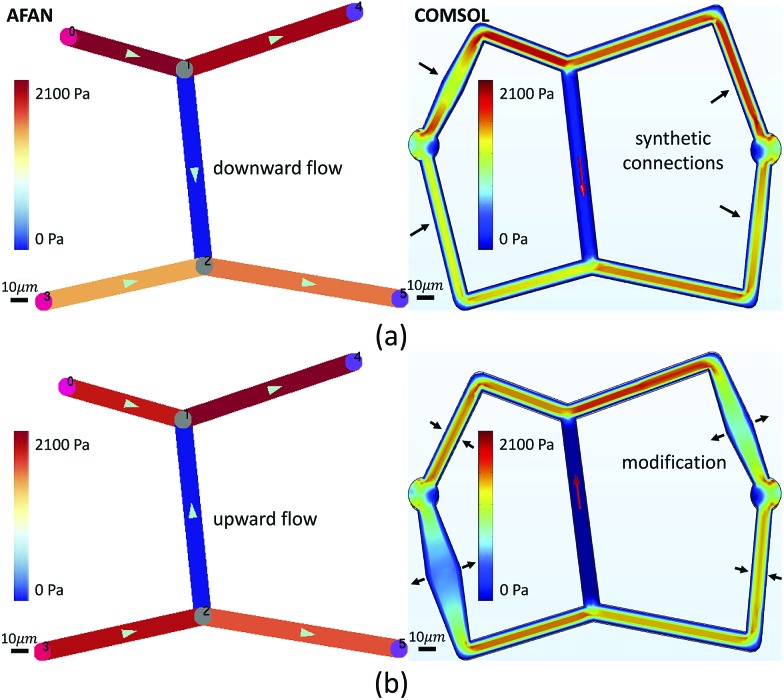
Validation of the flow-controlling capability of our method using a Wheatstone bridge planar network. A blue-red Brewer color map is used to visualize the flow velocity of each channel. The AFAN results closely match COMSOL results in predicting the flow direction throughout the ROI network. (a) The augmented network is designed to have downward flow in the central channel. (b) Augmented connections are modified to induce upward flow in the central channel. (a and b) SB = 10 μm.

We first designed a simple planar network based on the Wheatstone bridge structure which allows for control of the flow direction in the central channel based on varying boundary conditions. We used AFAN to specify two sets of boundary conditions with inverse flow directions in the central channel *via* different augmented connections (Fig. 5). We imported the resulting volumes into COMSOL (; https://www.comsol.com) to simulate a laminar flow using the same boundary setups. The comparison shows consistency of flow directions between two results, demonstrating our ability to control the flow profile within the ROI network *via* synthetic connections, or augmentation channels.

**Fig. 6:**
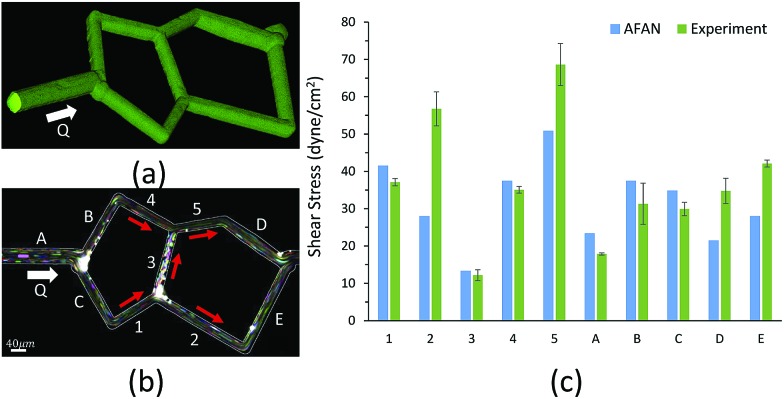
Microfluidic validation of the AFAN method based on an SMN. The designed network is fabricated *via* laser-induced degradation of a PEGDA hydrogel polymerized in a microfluidic housing. (a) A 3D rendering of the fabricated network shows circular cross-sections filled with 2000 kDa FITC-labeled dextran. (b) A 2D projection image composed of 988 time-lapse images of the microfluidic network depicts flowing 3 μm polystyrene particles, presented as colored streaks, that are used for particle image velocimetry. White lines indicate channel boundaries, determined using 2000 kDa FITC-labeled dextran. (c) The wall shear stress in each segment was calculated based on the average flow velocity and compared to the simulation results using AFAN, where the error bars represent standard deviation. (b) SB = 40 μm.

After fabrication, analysis of FITC-dextran images (Fig. 6a) showed a close agreement in vessel diameters between the mask and fabricated network as previously demonstrated.^31^ Segment diameters range from 18.2 μm to 19.2 μm in segments 1–5 of the fabricated network, and 19.2 μm to 30.6 μm in segments A–E; note that segments A and D were purposefully set larger than the rest of the segments. Segment diameters are 20.0 μm in segments 1–5 of the original network; 30.0 μm in segment A, 20.0 μm in segment B, 22.0 μm in segment C, 26.0 μm in segment D, and 20.0 μm in segment E; this shows a close agreement in network architecture between the fabricated network and its model counterpart. The measured velocity and calculated wall shear stress in all of the segments closely matched the AFAN simulation results (Fig. 6c). Segment 2 shows a slightly higher value than the simulation; this is likely due to particle lodging in the bifurcation between segments 1, 2, and 3 that causes a slight narrowing at the inlet of segment 2. Nevertheless, this experiment demonstrates that our AFAN software can be used to design and model physiologically relevant microfluidic networks. Here, we are referring to relevance with respect to wall shear stress that averaged 45 dynes per cm^2^ in the synthetic network which closely matches measured *in vivo* values for capillaries.^55^

### Three-dimensional, augmented, biomimetic network

**Fig. 7:**
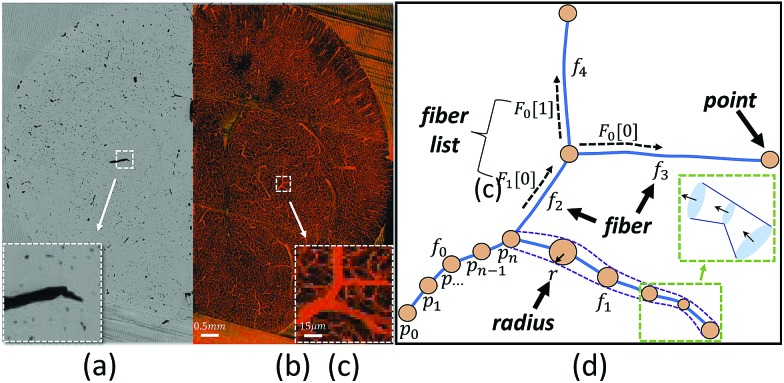
Mouse brain microvasculature reconstructed from 1000 KESM sections. (a) A raw section (*x*–*y* image) is shown, with microvascular cross-sections stained black using an India-ink perfusion. An inset shows a close-up of the India-ink embedded sample, in which vessels are stained black. (b) A maximum intensity projection of all 1000 KESM sections shows the overall structure of the sample. (c) An inset shows a viable ROI containing several inlets and outlets problematic for modeling. (d) Our approach represents the ROI network as a graph-like model with geometric components including points (orange dots), fibers (blue lines), and radii (dashed purple lines). The fiber lists *F*_0_ and *F*_1_ associated with each pivot point store fiber connectivity (*e.g.* a fiber in *F*_0_ has geometry specified outward from the given point). An inset shows visualizing fibers as a series of truncated generalized cones (TGCs) in AFAN. (b) SB = 0.5 mm. (c) SB = 15 μm.

Microvascular networks are difficult to accurately reconstruct due to the high spatial resolution and large volumes required (Fig. 7b). Microvessels within these networks are often less than 10 μm in diameter (Fig. 7c),^62^ however large volumes are necessary for understanding connectivity patterns and identifying the desired ROIs within microvascular beds.^63^ Images were therefore acquired using knife edge scanning microscopy (KESM)^46^ at a resolution of 0.6 μm laterally and 1.0 μm axially, which is sufficient to resolve the smallest microvessels.^47^ This method provides whole organ images at high contrast, making them easier to reconstruct. Vessel center lines and radii were extracted using an automated predictor-corrector algorithm^49^ that reconstructs the medial axis of each capillary fiber. This created an explicit graph model storing the architecture of the segmented network (Fig. 7d) and provided an accurate description of connectivity for flow simulations.

**Fig. 8:**
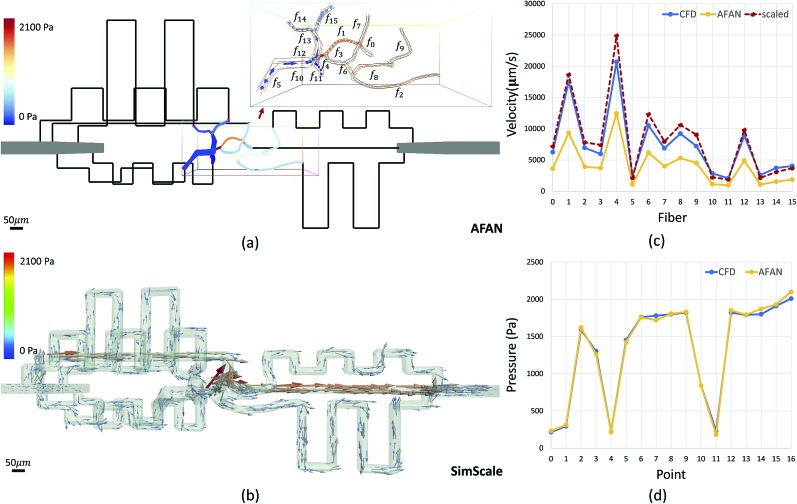
CFD validation of the AFAN method based on an RMN. (a) The simulation and visualization of laminar flow through a designed network using AFAN. Synthetic connections are rendered as black lines to reduce the memory usage for better visualization of the ROI network. An inset shows the velocity field across the ROI and its associated vessel segment numbering. (b) Visualizing and analyzing the CFD model using ParaView. The velocity field is visualized using arrow glyphs color-mapped by the flow velocity. (c) A velocity comparison between AFAN and SimScale simulations. Dashed red line represent maximum velocity values computed from AFAN average velocities values. (d) A pressure comparison shows great consistency between two methods. (a–b) SB = 50 μm.

The average velocity of each channel was initially estimated using AFAN (Fig. 8a) while the maximum velocity of each channel was calculated using SimScale (Fig. 8b). The comparison shows that our linear method succeeds in quantifying the velocity and pressure fields across the augmented network (Fig. 8c and d). It also shows that the deviation between the average flow velocity and maximum velocity can be compensated by a scaling factor, which is equal to 2 for circular channels (Fig. 8c). This is the result of integrating the velocity profile along the channel cross-section.

**Fig. 9:**
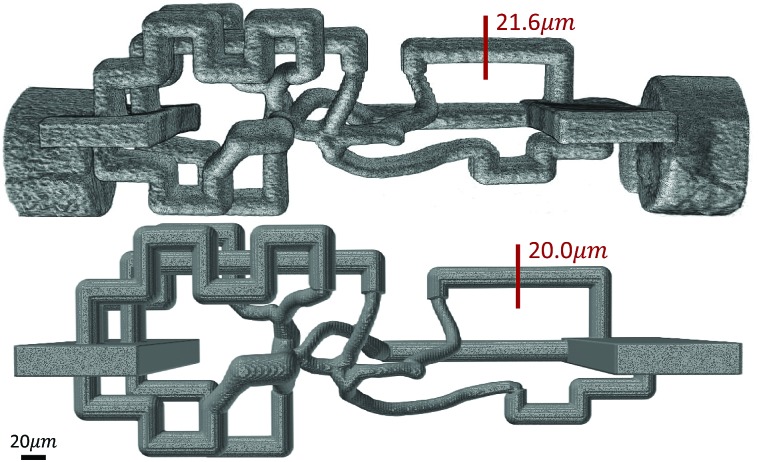
3D volume rendering of a fabricated microvascular network (top) and its digital mask (bottom) shows a close agreement in overall architecture and channel diameters between them. SB = 20 μm.

An RMN with vessel diameters ranging from 10–20 μm (Fig. 2) was also designed and fabricated. A 3D volume rendering of the fabricated network (Fig. 9) was created using Amira (ThermoFisher Scientific), which demonstrates a great agreement in network structure between the mask and fabricated network.

## Conclusion

In this paper, we proposed a new method for designing microvascular networks to enforce controlled flow when fabricated in microfluidic devices. To fulfill this aim, we developed software for simulating, characterizing, and visualizing microvascular networks for *in vitro* applications. We demonstrated the flow predictability of our linear model using rigorous CFD methods. We also demonstrated the viability of our network augmentation technique by fabricating an AFAN-developed microfluidic device and tracking fluid flow. The predicted network characteristics, including the structure, fluid velocity, and wall shear stress, closely match experimental results. This opens the door to creating microfluidic models of microvascular networks whose structure and fluid flow parameters mimic that of their *in vivo* counterparts. This will enhance the use of *in vitro* cell culture models by allowing researchers to more closely recapitulate *in vivo* flow patterns. Ideally, we can bridge different sub-networks in series to study blood circulation between different regions or across whole organs, such as the brain.^64^,^65^ AFAN has been integrated into an open-source application available online (; https://www.stim.ee.uh.edu). However, this current approach is based on the assumption of Newtonian flow, and thus limited to microvessels with high shear rates. To fully represent realistic *in vivo* flow, we plan to explore future modifications to our algorithm that will account for non-Newtonian effects, as well as validate our results using more accurate flow media.

## Authors' contributions

Jiaming Guo implemented the AFAN software, optimized the network augmenting method, and conducted CFD-based simulations. Pavel Govyadinov segmented and reconstructed the microvascular data. Keely Keller and John Slater fabricated the microfluidic networks and conducted the particle tracking experiments. Paul Ruchhoeft and David Mayerich developed the theoretical model. All authors reviewed and contributed to the final manuscript.

## Conflicts of interest

There are no conflicts to declare.

## References

[cit1] Song H. H. G., Rumma R. T., Ozaki C. K., Edelman E. R., Chen C. S. (2018). Cell Stem Cell.

[cit2] Tien J. (2014). Curr. Opin. Chem. Eng..

[cit3] Hasan A., Paul A., Vrana N. E., Zhao X., Memic A., Hwang Y.-S., Dokmeci M. R., Khademhosseini A. (2014). Biomaterials.

[cit4] Miller J. S. (2014). PLoS Biol..

[cit5] Moya M. L., Hsu Y. H., Lee A. P., Hughes C. C., George S. C. (2013). Tissue Eng., Part C.

[cit6] Whisler J. A., Chen M. B., Kamm R. D. (2012). Tissue Eng., Part C.

[cit7] Ehsan S. M., Welch-Reardon K. M., Waterman M. L., Hughes C. C. W., George S. C. (2014). Integr. Biol..

[cit8] Kim S., Lee H., Chung M., Li Jeon N. (2013). Lab Chip.

[cit9] Park Y. K., Tu T.-Y., Lim S. H., Clement I. J. M., Yang S. Y., Kamm R. D. (2014). Cell. Mol. Bioeng..

[cit10] Cuchiara M. P., Gould D. J., McHale M. K., Dickinson M. E., West J. L. (2012). Adv. Funct. Mater..

[cit11] Moon J. J., Saik J. E., Poché R. A., Leslie-Barbick J. E., Lee S.-H., Smith A. A., Dickinson M. E., West J. L. (2010). Biomaterials.

[cit12] Chen M. B., Whisler J. A., Fröse J., Yu C., Shin Y., Kamm R. D. (2017). Nat. Protoc..

[cit13] Chen M. B., Whisler J. A., Jeon J. S., Kamm R. D. (2013). Integr. Biol..

[cit14] Alonzo L. F., Moya M. L., Shirure V. S., George S. C. (2015). Lab Chip.

[cit15] Ghaemmaghami A. M., Hancock M. J., Harrington H., Kaji H., Khademhosseini A. (2012). Drug Discovery Today.

[cit16] Prabhakarpandian B., Shen M.-C., Nichols J. B., Garson C. J., Mills I. R., Matar M. M., Fewell J. G., Pant K. (2015). J. Controlled Release.

[cit17] Chen Y. C., Chen G. Y., Lin Y. C., Wang G. J. (2010). Microfluid. Nanofluid..

[cit18] Wong K. H. K., Chan J. M., Kamm R. D., Tien J. (2012). Annu. Rev. Biomed. Eng..

[cit19] Forouzan O., Yang X., Sosa J. M., Burns J. M., Shevkoplyas S. S. (2012). Microvasc. Res..

[cit20] Zheng Y., Chen J., Craven M., Choi N. W., Totorica S., Diaz-Santana A., Kermani P., Hempstead B., Fischbach-Teschl C., López J. A., Stroock A. D. (2012). Proc. Natl. Acad. Sci. U. S. A..

[cit21] Jeon J. S., Bersini S., Gilardi M., Dubini G., Charest J. L., Moretti M., Kamm R. D. (2015). Proc. Natl. Acad. Sci. U. S. A..

[cit22] Buchanan C. F., Voigt E. E., Szot C. S., Freeman J. W., Vlachos P. P., Rylander M. N. (2013). Tissue Eng., Part C.

[cit23] Jeon J. S., Zervantonakis I. K., Chung S., Kamm R. D., Charest J. L. (2013). PLoS One.

[cit24] Qiu Y., Ahn B., Sakurai Y., Hansen C. E., Tran R., Mimche P. N., Mannino R. G., Ciciliano J. C., Lamb T. J., Joiner C. H., Ofori-Acquah S. F., Lam W. A. (2018). Nat. Biomed. Eng..

[cit25] Doraiswamy A., Narayan R. J. (2010). Philos. Trans. R. Soc., A.

[cit26] Miller J. S., Stevens K. R., Yang M. T., Baker B. M., Nguyen D.-H. T., Cohen D. M., Toro E., Chen A. A., Galie P. A., Yu X., Chaturvedi R., Bhatia S. N., Chen C. S. (2012). Nat. Mater..

[cit27] He J., Zhu L., Liu Y., Li D., Jin Z. (2014). J. Mater. Sci.: Mater. Med..

[cit28] Kolesky D. B., Truby R. L., Gladman A. S., Busbee T. A., Homan K. A., Lewis J. A. (2014). Adv. Mater..

[cit29] Therriault D., White S. R., Lewis J. A. (2003). Nat. Mater..

[cit30] Wu W., DeConinck A., Lewis J. A. (2011). Adv. Mater..

[cit31] Heintz K. A., Bregenzer M. E., Mantle J. L., Lee K. H., West J. L., Slater J. H. (2016). Adv. Healthcare Mater..

[cit32] Heintz K. A., Mayerich D., Slater J. H. (2017). J. Visualized Exp..

[cit33] Pradhan S., Keller K. A., Sperduto J. L., Slater J. H. (2017). Adv. Healthcare Mater..

[cit34] DeForest C. A., Anseth K. S. (2011). Nat. Chem..

[cit35] Tibbitt M. W., Kloxin A. M., Dyamenahalli K. U., Anseth K. S. (2010). Soft Matter.

[cit36] Applegate M. B., Coburn J., Partlow B. P., Moreau J. E., Mondia J. P., Marelli B., Kaplan D. L., Omenetto F. G. (2015). Proc. Natl. Acad. Sci. U. S. A..

[cit37] Ilina O., Bakker G. J., Vasaturo A., Hoffman R. M., Friedl P. (2011). Phys. Biol..

[cit38] Kloxin A. M., Kasko A. M., Salinas C. N., Anseth K. S. (2009). Science.

[cit39] Sarig-Nadir O., Livnat N., Zajdman R., Shoham S., Seliktar D. (2009). Biophys. J..

[cit40] Brandenberg N., Lutolf M. P. (2016). Adv. Mater..

[cit41] Rosano J. M., Tousi N., Scott R. C., Krynska B., Rizzo V., Prabhakarpandian B., Pant K., Sundaram S., Kiani M. F. (2009). Biomed. Microdevices.

[cit42] Prabhakarpandian B., Pant K., Scott R. C., Patillo C. B., Irimia D., Kiani M. F., Sundaram S. (2008). Biomed. Microdevices.

[cit43] Oshima M., Torii R., Kobayashi T., Taniguchi N., Takagi K. (2001). Comput. Meth. Appl. Mech. Eng..

[cit44] Freund J. B. (2014). Annu. Rev. Fluid Mech..

[cit45] Culver J. C., Hoffmann J. C., Poché R. A., Slater J. H., West J. L., Dickinson M. E. (2012). Adv. Mater..

[cit46] Mayerich D., Abbott L., McCormick B. (2008). J. Microsc..

[cit47] Mayerich D., Kwon J., Sung C., Abbott L., Keyser J., Choe Y. (2011). Biomed. Opt. Express.

[cit48] Chung J. R., Sung C., Mayerich D., Kwon J., Miller D. E., Huffman T., Abbott L. C., Keyser J., Choe Y. (2011). Front. Neuroinf..

[cit49] Govyadinov P. A., Womack T., Chen G., Eriksen J., Mayerich D. (2018). IEEE Trans. Visual. Comput. Graph..

[cit50] Larina I. V., Sudheendran N., Ghosn M. G., Jiang J., Cable A., Larin K. V., Dickinson M. E. (2008). J. Biomed. Opt..

[cit51] Gatehouse P. D., Keegan J., Crowe L. A., Masood S., Mohiaddin R. H., Kreitner K.-F., Firmin D. N. (2005). Eur. Radiol..

[cit52] Page M., Beaudoin M., Giroux A.-M. (2011). International Journal of Fluid Machinery and Systems.

[cit53] Ayachit U., The ParaView Guide: A Parallel Visualization Application, Kitware Inc., 2015.

[cit54] Kumar D., Vinoth R., Adhikari R., Vijay Shankar C. (2017). Biomed. Res..

[cit55] Papaioannou T. G., Stefanadis C. (2005). Hellenic J. Cardiol..

[cit56] Young D. F., Munson B. R., Okiishi T. H. and Huebsch W. W., A Brief Introduction To Fluid Mechanics, John Wiley & Sons, 2010.

[cit57] Oh K. W., Lee K., Ahn B., Furlani E. P. (2012). Lab Chip.

[cit58] Liu R., Jiang Y., Li B., Yu L. (2016). Microfluid. Nanofluid..

[cit59] Reichold J., Stampanoni M., Lena Keller A., Buck A., Jenny P., Weber B. (2009). J. Cereb. Blood Flow Metab..

[cit60] Saggiomo V., Velders A. H. (2015). Adv. Sci..

[cit61] Mayerich D. M. and Keyser J., Proceedings of the 2008 ACM symposium on Solid and physical modeling, 2008, pp.pp. 353–358353–358.

[cit62] Hu J., Cao Y., Wu T., Li D., Lu H. (2014). Med. Phys..

[cit63] Cassot F., Lauwers F., Lorthois S., Puwanarajah P., Cances-Lauwers V., Duvernoy H. (2010). Brain Res..

[cit64] Blinder P., Tsai P. S., Kaufhold J. P., Knutsen P. M., Suhl H., Kleinfeld D. (2013). Nat. Neurosci..

[cit65] Kleinfeld D., Bharioke A., Blinder P., Bock D. D., Briggman K. L., Chklovskii D. B., Denk W., Helmstaedter M., Kaufhold J. P., Lee W.-C. A., Meyer H. S., Micheva K. D., Oberlaender M., Prohaska S., Reid R. C., Smith S. J., Takemura S., Tsai P. S., Sakmann B. (2011). J. Neurosci..

